# Lentil and Fava Bean With Contrasting Germination Kinetics: A Focus on Digestion of Proteins and Bioactivity of Resistant Peptides

**DOI:** 10.3389/fpls.2021.754287

**Published:** 2021-10-25

**Authors:** Sara Bautista-Expósito, Albert Vandenberg, Elena Peñas, Juana Frias, Cristina Martínez-Villaluenga

**Affiliations:** ^1^Department of Food Characterization, Quality and Safety, Institute of Food Science, Technology and Nutrition (ICTAN-CSIC), Madrid, Spain; ^2^Department of Plant Sciences, College of Agriculture and Bioresources, University of Saskatchewan, Saskatoon, SK, Canada

**Keywords:** seed imbibition, germination kinetics, peptidome analysis, bioactivity, lentil, fava bean, digestion, proteins

## Abstract

Germination offers advantages to improve legume protein digestibility as it disintegrates seed structure and hydrolyzes proteins and anti-nutrients. Seed permeability (related to polyphenol content of seed coats) is an important factor affecting the duration of seed germination and its impact on protein digestibility and bioactivity. The objective was to compare the effect of seed germination on protease activity, structure, and proteolysis of four selected legumes with contrasting seed coat polyphenol profiles (gray zero-tannin lentil [GZL], beluga lentil [BL], and dehulled red lentil [DL]; and zero tannin/low vicine–convicine fava bean [ZF]). Protein hydrolysis was characterized during germination and digestion with respect to proteins, peptides, and free amino acids (FAAs). *In vitro* antihypertensive and antioxidant activities of digests were investigated, and the peptidomic characterization [high-performance liquid chromatography coupled to tandem mass spectrometry (HPLC-MS/MS)] and identification of bioactive fragments in intestinal digests were performed. Regardless of the seed type, germination increased protease activity and reduced the levels of phytic acid, trypsin inhibitors, and tannins (only in BL). A significant proteolysis of the 7S and 11S globulins and a concomitant increase of peptides and FAAs were observed in all sprouted legumes. Digestion kinetics in sprouts revealed a faster generation of FAAs and peptides than in dry seeds, with changes being more evident for DL, associated with a faster imbibition, germination, and sprout growth. In contrast, BL sprouts showed the lowest protein digestibility, likely due to a lower protease activity, seed structure disintegration, and higher anti-nutrient levels in comparison to GZL, DL, and ZF. Moreover, the digestion of sprouts resulted in a higher number of resistant peptides in DL and ZF that matched with previously reported bioactive sequences, suggesting a promising health potential of legume sprouts that was confirmed *in vitro*. The results suggested that the germination process improved protein digestibility and the health-promoting potential of lentil and fava bean proteins although these changes were more evident in DL due to its rapid imbibition, faster germination, and sprout development. This study will provide important information for either plant breeders to develop legume varieties with permeable seed coats or food producers that could use dehulled seeds for efficient production of sprouts as sustainable food sources of plant proteins with improved nutritional and healthy properties.

## Introduction

Protein is one of the major food components for balanced human diets. There is an increasing interest in exploring plant-based protein sources to encourage their consumption as an alternative to animal proteins motivated by the global awareness on food security, environmental sustainability, and health benefits (Loveday, 2019). Among plant protein sources, legume crops are the main dietary sources for >1 billion people, providing high protein yield (18–32%) and generous economic and environmental benefits (Saricaoglu, 2020; Rahate et al., 2021). Legume crops provide symbiotically fixed nitrogen, thus reducing the contribution to atmospheric greenhouse gases and improving soil fertility for successive crops.

Some pulse crops (pea, fava bean, and lentil) are excellent sources of high-quality proteins for human nutrition. In general, legume proteins contain all the essential amino acids (EAA) and are rich in lysine (K), leucine (L), aspartic acid (D), glutamic acid (E), and arginine (R); whereas cysteine (C), methionine (M), tryptophan (W), and threonine (T) are considered to be limited (Boye et al., 2010; Joshi et al., 2017). Relatively little information is available in the literature regarding the behavior of legume proteins during human digestion and the characterization of the resulting products of protein hydrolysis. Knowledge on how plant proteins behave in the human gut and their associated nutritional value will eventually contribute to the development of high-quality and healthier plant-based food products.

It is known that legume protein digestibility and the bioavailability of amino acids are lower in comparison with animal proteins (Ohanenye et al., 2020). The seed structure, protein primary structure, and the presence and quantity of anti-nutrients (trypsin and chymotrypsin inhibitors, tannins, and phytic acid) are the major determinants of seed-based protein quality. Food processing techniques involving the changes of seed structure, removal of anti-nutrients as well as solubilization, fractionation, and concentration of plant proteins may improve legume protein quality. Germination is an ancient sustainable bioprocess that provides a great opportunity for improving legume protein digestibility as it involves enzymatic hydrolysis that modifies the seed structure, resulting in the breakdown of proteins stored within plant cell vacuoles into free amino acids (FAAs) (Sjöö and Nilsson, 2017; Chen et al., 2019) and reducing anti-nutrient content (Nosworthy et al., 2018). Nevertheless, the behavior of proteins during human gastrointestinal digestion of sprouted legumes in terms of the distribution of nitrogen fraction and identification of hydrolysis products remains unknown. Improved digestibility of proteins in sprouted legumes may concomitantly facilitate the release of bioactive peptides, providing additional health benefits (Ohanenye et al., 2020).

The seed coat not only has protective and structural functions but also provides a crucial role in timing of the seed germination of legume crops by regulating water uptake (Smýkal et al., 2014). Despite not being formally demonstrated, the main testa pigments, proanthocyanidins, are hypothesized to play a role in seed coat permeability (Nakayama and Komatsu, 2008; Smýkal et al., 2014). The hydrophobic nature of pigmented seed coats, likely due to the presence of proanthocyanidins (condensed tannins) in the epidermis, was presumed to be responsible for the delayed permeability of seeds and germination. The modification of seed permeability to water through the reduction of tannin content of seed coats may provide additional benefits in legume protein digestibility based on a reduced anti-nutrient content and speeding up the germination-induced mobilization of seed storage proteins. Evaluating specific legume crops with known genetically based contrasting seed coat biochemical profiles could identify genetic variability in the effect of their germination on the quality and the health-promoting potential of the protein fraction. The objective was to study a variation in the kinetics of imbibition, radicle emergence, and sprout elongation of the selected legume types representing contrasting seed permeability (dehulled, zero, low, and high tannin seeds) to establish associations with differences in germination-induced changes on the anti-nutrient and protein content, amino acid profile, proteolysis, cotyledon cellular microstructure, and their influence on *in vitro* protein digestibility and bioactivity. Kinetics and yields of the protein breakdown products and, for the first time, the peptidome generated by legume flours at the end of intestinal phase of digestion and the identification of bioactive peptides are presented. This study will provide important information for either plant breeders to develop legume varieties with permeable seed coats or food producers that could use seeds with improved permeability for efficient production of sprouts as sustainable food sources of plant proteins with improved nutritional and healthy properties.

## Materials and Methods

### Materials and Seed Germination

Legume seed types representing low tannin [gray zero-tannin lentil (GZL) breeding line from the University of Saskatchewan (UoS), generically related to the cultivar CDC Gold (GZL) and low tannin/low vicine–convicine fava bean (ZF) breeding line 951-1-11] and high tannin/anthocyanin [Indianhead lentil, a cultivar developed at UoS marketed as beluga lentil (BL)] were selected to sample seed coat biochemical diversity. An additional seed type representing a decorticated legume sample [dehulled commercial sample of CDC Maxim red lentil from UoS (DL)] was also selected for additional comparison. All seed types were stored at 4°C in vacuum-sealed plastic bags. For germination trials, 150 g of each seed type were disinfected for 10 min with 0.5% sodium hypochlorite before soaking in sterile water (1 : 6, w : v) at 20°C and then rinsed three to four times with tap water. Soaking time was established for each seed type by plotting the timeline of seed weight during the imbibition phase. Soaked seeds were covered by a moist filter paper and placed in a thermostatically controlled climatic chamber (model EC00-065, Snijders Scientific, Tilburg, The Netherlands), with a water circulating system to maintain air humidity ≥90%. Sprout growth was monitored over time measuring the length of rootless and shoots. Germination trials were performed at 20°C at two endpoints (96 and 144 h) in the dark. Under these experimental conditions, the germination rate reached values over 90% in all the cases (Supplementary Table 1). Samples were immediately frozen at −80°C and freeze-dried using a lyophilizer (Virtis Company, Inc., Gardiner, NY, USA). Flours from dry seeds and sprouts were obtained using an Ika M20 laboratory mill (IKA-Werke, Staufen, Germany). After milling, flours were sieved (mesh size 300 μm) and stored at −20°C in vacuum-sealed plastic bags.

### *In vitro* Simulated Gastrointestinal Digestion

Flours were digested using INFOGEST 2.0 protocol of static *in vitro* gastrointestinal digestion *(*Brodkorb et al., 2019). First, 1.25x stocks of simulated salivary fluid (SSF), simulated gastric fluid (SGF), and simulated intestinal fluid (SIF) were prepared as given in detail in the INFOGEST 2.0 method. Raw and germinated flours (2 g) were dispersed in 2 ml of SSF containing 75 U/ml of human salivary amylase (E.C.3.2.1.1, Sigma-Aldrich, St. Louis, MO, USA), and then incubated for 2 min at 37°C and pH 7. The oral digest was immediately incubated with SGF (50:50, v:v) containing 2,000 U/ml of pepsin porcine from gastric mucosa (E.C. 3.4.23.1, Sigma-Aldrich, St. Louis, MO, USA) at pH 3 and 37°C for 2 h in an orbital shaker. Gastric digestion was stopped for adjusting pH at 7 with NaOH 1 M. The intestinal phase was initiated by the addition of SIF (50:50, v:v) containing 100 U/ml of pancreatin from porcine pancreas (Sigma-Aldrich, St. Louis, MO, USA) and porcine bile extract (2.5 mM final volume) for 2 h at 37°C at pH 7. The intestinal phase was stopped by thermal treatment at 80°C for 10 min. Gastric (g) and intestinal (i) digests were freeze-dried and stored at −20°C in vacuum-sealed plastic bags.

### Determination of Anti-nutrients

Trypsin inhibitory activity (TIA) was determined as described previously by Sueiro et al. (2015). Flours (100 mg) were dispersed in 5 ml of 0.01 M NaOH (pH 8.4–10.0) and incubated for 3 h at 20°C in a Thermomixer C (Eppendorf, Thermo Fisher Scientific, Waltham, MA, USA). The final volume of the resulting solution was adjusted to 10 ml with distilled water, shaken, and left standing for 15 min, after which aliquots of 1 ml were withdrawn and diluted to cause 40–60% inhibition of the trypsin activity. TIA was expressed as trypsin inhibitory units (TIU)/mg of flour.

Phytic acid content was determined by the Phytic Acid (Phytate)/Total Phosphorus Assay kit (Megazyme, Wicklow, Ireland). Phytic acid content was expressed as mg/g flour.

An analysis of condensed tannins was carried out as described previously by Zurita et al. (2012). Briefly, 200 mg of flour were hydrolyzed with 10 ml of hydrochloric acid (HCl)/n-butanol (5:95, v:v) containing 0.7 g/L of iron (III) chloride at 100°C for 1 h. Samples were centrifuged (14,000 × *g* for 10 min) and supernatants were washed two times with 10 ml of BuOH:HCl:FeCl_3_. After adjusting the final volume to 25 ml, absorbance was measured at 550 nm in a Synergy HT microplate reader (BioTek Instruments, Winooski, VT, USA). Delphinidin chloride (processed in the same way as samples) was used as standard (Extrasynthese, Genay, France). External calibration curve in the linear range from 0 to 40 μg/ml was plotted to calculate condensed tannin content. The values were expressed in mg/g of flour.

### Determination of Protein, Peptides, and Amino Acids

The total protein content in dry seeds and germinated legume flours (0.5 g) was determined by the Dumas method using a Trumac nitrogen analyzer (Leco Corporation, St Joseph, MI, USA). Nitrogen content was transformed using a conversion factor of 6.25 and data were expressed as g protein/100 g flour.

Molecular weight (MW) distribution of peptides (<3, 3–10, and 10–30 kDa) in the total protein fraction was measured by the Quantitative Colorimetric Peptide Assay kit (Pierce, Thermo Fisher Scientific, Waltham, MA, USA) in permeates obtained by ultrafiltration through Amicon units with the cellulose membranes of 3, 10, and 30 kDa MW cutoff (Merck, Darmstadt, Germany). Absorbance was read at 480 nm using a Synergy HT microplate reader (BioTek Instruments, Winooski, VT, USA). The results were expressed as g/100 g of flour or g/100 g of protein for gastric and intestinal digests.

Total amino acids (TAAs) were determined in flours after acid hydrolysis using 6 N HCl at 110°C under vacuum for 22 h containing 100 μM of internal standard (Norleucine, Sigma-Aldrich, St. Louis, MO, USA). FAAs were extracted by dispersing 100 mg of flours or freeze-dried digests in 1 ml of 0.1 N HCl for 1 h at 4°C. The purification, derivatization, and analysis of TAAs and FAAs were performed using the EZ:faast™ GC-FID Physiological (Free) Amino Acids (Phenomenex, Torrance, CA, USA) in an Agilent gas chromatograph 7820 A (Agilent, Santa Clara, CA, USA) equipped with a flame ionization detector (FID), an automatic injector, and a Zebron ZBAAA capillary column (10 m x 250 μm x 0.25 μm; Phenomenex, Torrance, CA, USA). Initially, the temperature of the oven was set at 110°C, and the temperature increased using a ramp of 32°C/min up to 320°C. Derivatized samples were injected (2 μl) in a split mode (15:1) at 250°C. The FID detector was set to 320°C, and the carrier helium gas flow rate was maintained at 1.5 ml/min. External calibration was carried out using a multistandard solution of 26 amino acids, and the results were corrected according to the internal standard recovery. TAA profile was expressed as % of TAAs. FAA content was expressed as g/100 g of flour or g/100 g of protein for gastric and intestinal digests.

### Protease Activity and Degree of Hydrolysis

Protease activity was determined in dry seeds and germinated legume flours. Protein extracts were obtained by an incubation of 200 mg of flour in 1 ml of double distilled water at 25°C and 1,500 rpm of agitation speed for 60 min using a Thermomixer C orbital shaker (Eppendorf, Thermo Fisher Scientific, Waltham, MA, USA). After incubation, the samples were centrifuged at 14,000 *g*, 25°C for 10 min. The Fluorescent Protease Assay kit (Pierce, Thermo Fisher Scientific, Waltham, MA, USA) was used for evaluating total protease activity in protein extracts. Fluorescence was read in a microplate reader (Biotek Instruments, Winooski, VT, USA) at excitation and emission wavelengths of 485 and 538 nm, respectively. The increase of relative fluorescence units within 10 min of reaction was plotted as a function of trypsin (standard) concentration. Different concentrations of trypsin ranging from 0 to 1,500 ng/ml (in distilled water) were used to plot an external calibration curve. The results were expressed as ng of trypsin activity equivalents/mg of flour.

The degree of hydrolysis (DH) was monitored using the reported *o*-phthaldialdehyde (OPA) spectrophotometric assay in 96-well plates (Nielsen et al., 2001). Briefly, 50 mg of flour were dispersed in 50 ml of bidistilled water and centrifuged at 10,000 *g* for 5 min at 23°C. Supernatant (30 μl) was placed into each well and mixed in 225 μl of OPA reagent (Sigma-Aldrich, St. Louis, MO, USA). After incubation at 23°C for 2 min, the absorbance was measured at 340 nm using a Synergy HT microplate spectrophotometer (BioTek Instruments, Winooski, VT, USA). Serine was used as a standard at the concentration of 0.1 mg/ml of deionized water (0.9516 meqv/L). The DH was calculated based on the total number of peptide bonds per protein equivalents and the number of hydrolyzed bonds according to theoretical general values (Nielsen et al., 2001).


$$\begin{array}{l} \text{DH (\%)}=h/h_{\text{tot}}\;*\;100 \end{array}$$

where *h*_tot_ was 8.0 and *h* was expressed as


$$\begin{array}{l} h=\text{Ser-N}{\text{H}}_{2}-\beta /\text{αmeqv}/\text{g protein} \end{array}$$

Here, *β* was 0.40, α was 1.00, and Ser-NH_2_ was determined as


$$\begin{array}{l} {\text{Ser-NH}}_{2}={\text{ Abs}}_{\text{sample}}-{\text{ Abs}}_{\text{blank}}{\text{/Abs}}_{\text{standard}}-{\text{ Abs}}_{\text{blank}}\\ \;*\text{0}.\text{9516 meqv/L }*\text{ 0}.\text{1 }*\text{ 100/}\;\text{X} \end{array}$$

where X was a 0.05-g sample and 0.05 was the sample volume in liter.

### Sodium Dodecyl Sulfate-Polyacrylamide Gel Electrophoresis

Soluble protein extracts obtained from flours were diluted (1:4, v:v) in NuPAGE® dodecyl lithium sulfate sample buffer (Invitrogen, Thermo Fisher Scientific, Waltham, MA, USA) and analyzed by sodium dodecyl sulfate-polyacrylamide gel electrophoresis (SDS-PAGE). After heating at 70°C for 10 min, 20 μg protein/well were loaded on NuPAGE® Novex 4–12% Bis-Tris gels (Invitrogen, Thermo Fisher Scientific, Waltham, MA, USA), and run in a Mini Gel Tank (Invitrogen, Thermo Fisher Scientific, Waltham, MA, USA) with NuPAGE® 2-(N-morpholino) ethanesulfonic acid-SDS (Invitrogen, Thermo Fisher Scientific, Waltham, MA, USA) at 200 V for 35 min. Novex® Sharp Prestained Protein Standard (Invitrogen, Thermo Fisher Scientific, Waltham, MA, USA) containing polypeptides from 3.5 to 260 kDa was used as an MW marker.

In digestion experiments, extracts were diluted in Tricine SDS sample buffer (Invitrogen, Thermo Fisher Scientific, Waltham, MA, USA) and loaded (13 μg protein/well) onto Novex 16% Tricine gels (Invitrogen, Thermo Fisher Scientific, Waltham, MA, USA). Electrophoretic separation was performed for 65 min at 125 V. PageRuler^TM^ Unstained Low Range Protein Ladder (Invitrogen, Thermo Fisher Scientific, Waltham, MA, USA) containing polypeptides from 3.4 to 100 kDa was used as the MW standard. Gels were stained with SimplyBlue SafeStain (Invitrogen, Thermo Fisher Scientific, Waltham, MA, USA) for 1 h and distained in deionized water for 2 h. Images were obtained using the ChemiDoc MP Imaging System (Bio-Rad, Hercules, CA, USA). Band optical density and MW were measured using the Quantity One software (Bio-Rad, Hercules, CA, USA).

### Transmission Electron Microscopy

Seeds that were imbibed and germinated for 96 h were selected for observation using the JEOL 1230 transmission electron microscope (Jeol Ltd., Tokyo, Japan). Seeds were dissected into several small pieces with a scalpel and fixed in 2.5% glutaraldehyde in phosphate buffer saline (PBS, pH 7.4) containing 1.5% *p*-formaldehyde for 2 h at 4°C under vacuum. Subsequently, the samples were imbibed in 5% glutaraldehyde in PBS containing 4% *p*-formaldehyde at room temperature for 2 h. After several washes with PBS, the seed tissues were dehydrated gradually using ethanol solutions from 30 to 100% followed by Spurr's resin infiltration at 4°C for 3 days. For each experimental group, a minimum of three seed replicates were examined under electron microscopy. Ultra-thin sections of embedded tissues were prepared using a diamond knife of a rotary microtome and collected on 200 mesh copper grids. The sections were double stained with 5% uranyl acetate and lead citrate for 20 and 2 min, respectively, at room temperature. The stained sections were examined in the transmission electron microscope at 80 kV, and the images were captured with a TVIPS 4x4K camera (Jeol Ltd., Tokyo, Japan).

### Bioactivity of Peptide Fractions

Angiotensin converting enzyme (ACE) inhibitory activity of peptide fractions <3 kDa was determined as previously reported in Garcia-Mora et al. (2014). Briefly, 50 μl of 3mU/ml of ACE from rabbit lung (Sigma-Aldrich, Madrid, Spain) was mixed with 200 μl of Abz-Gly-Phe (NO_2_)-Pro (Cymit Quimica, Barcelona, Spain) in the absence (control) or presence of diluted peptide fractions (50 *μ*l). Reactions were carried out in 96-well plates at 37°C for 30 min. Fluorescence was read every minute for 30 min at emission and excitation wavelengths of 335 and 405 nm, respectively, in a Synergy HT microplate reader (BioTek Instruments, Winooski, VT, USA). The sample concentration (in mg/ml) that inhibited 50% of enzyme activity (IC_50_) was calculated by plotting the nonlinear regression sigmoidal dose-response curves in GraphPad Prism 4.00 (GraphPad Software, Inc., San Diego, CA, USA).

The antioxidant activity was determined by the two different methods: the oxygen radical absorbance capacity (ORAC) and 2,2′-azino-bis-3-ethylbenzothiazoline-6-sulfonic acid (ABTS) radical scavenging assay (Martín-Diana et al., 2021). In the ORAC method, 180 μl of 70 nM fluorescein was mixed with 90 μl of 12 mM AAPH and 30 μl of diluted sample. Reaction mixtures were placed in a black 96-well plate (Fisher Scientific, Waltham, MA, USA) and the fluorescence was measured in a Synergy HT microplate reader (BioTek Instruments, Winooski, VT, USA) every minute at excitation and emission wavelengths of 485 and 520 nm, respectively. An external calibration curve using Trolox as standard in a linear concentration range from 0 to 160 μM was prepared from a freshly made 1 mM stock solution. The results were expressed as mM of Trolox equivalents (TE)/g of intestinal digest. In the ABTS assay, a stock ABTS solution was prepared by mixing a 7 mM aqueous ABTS solution with 2.45 mM K_2_O_8_S_2_ in a 1:1 (v/v) ratio. Then, the stock ABTS solution was diluted with phosphate buffer (75 mM, pH 7.4) to obtain a working solution with an absorbance value of 0.70 ± 0.02 at 734 nm. A volume of 20 μl of diluted samples was mixed with 200 μl of ABTS working solution in a 96-well microplate. The absorbance was measured over 30 min at 734 nm in a Synergy HT microplate reader (BioTek Instruments, Winooski, VT, USA), and a Trolox calibration curve was used in the concentration range from 0 to 800 *μ*M. The results were expressed as mmol TE/g intestinal sample.

### Peptide Identification

For peptide identification, intestinal digests of seeds were analyzed by ultra-high performance liquid chromatography-tandem mass spectrometry (UPLC-MS/MS) using a nano Easy-nLC 1000 (Proxeon, Odense, Denmark), connected to an LTQ–Orbitrap Velos mass spectrometer (Thermo Scientific, Bremen, Germany) equipped with a nanoelectrospray ionization source. Lyophilized samples were suspended in 0.1% acetic acid until reaching the concentration of 0.5 mg/ml. Intestinal digests were purified using a C18 ZipTip (Millipore, Bellerica, MA, USA) reversed phase columns and eluted using 2% acetonitrile in water containing 0.1% formic acid. Sample aliquots of 4 μl were injected into the nLC-MS/MS equipment and desalted in Acclaim™ PepMap™ 100 precolumn and loaded onto a 0.075 mm x 500 mm, 2 μm bead Acclaim™ PepMap™ 100 C18 column (Thermo Fisher Scientific, Waltham, MA, USA) for analytical separation. Peptides were eluted using a gradient of 0.1% formic acid in water (solvent A) and 99.9% acetonitrile containing 0.1% formic acid (solvent B) at a flow rate of 250 nl/min. The mass spectra corresponding to the complete scan (m/z 400–2,000) were obtained with a resolution of 70,000, and the 15 most intense ions of each scan were selected for fragmentation by means of collision-induced dissociation in the ionic trap, with the collision energy normalized to 35%. Ions with single charge or no charge assignment were discarded. A dynamic exclusion was carried out with the duration of 20 s. Lentil and fava bean databases were prepared by combining the NCBI (https://www.ncbi.nlm.nih.gov) and Uniprot (https://www.uniprot.org) “*Lens*” and “*Vicia faba”* protein sequences. The peptide mass spectra were compared with protein database using Proteome Discoverer (version 1.4.1.14, Thermo Scientific, Waltham, MA, USA). Carbamidomethylation of cysteine and oxidation of methionine as fixed and dynamic modifications, and proteases trypsin and chymotrypsin (allowing 2 “miscleavages”) were selected in the search parameters. The selection of precursors and product ions was set to be tolerated at 10 ppm and 0.05 Da, respectively. The identification of the peptides was validated by the Percolator algorithm using the value of *q* ≤ 0.01 (Käll et al., 2007). The proteomics analysis was carried out in duplicate at the Proteomics and Genomics Facility of the Center for Biological Research (CIB-CSIC), a member of ProteoRed-ISCIII network. Each detected peptide was associated with its annotated sequence, intensity, parent protein accessions, and positions in the parent protein.

### Computational Analysis

Peptide patterns after intestinal digestion were represented by using the online software tool Peptigram (Manguy et al., 2017). The potential of peptides to be bioactive was predicted using the PeptideRanker tool (http://distilldeep.ucd.ie/PeptideRanker/) in the Discovery@Bioware server, and their theoretical bioactivity was expressed as the score values calculated (from 0 to 1, with 1 being the most likely to be bioactive). Moreover, the prediction of the bioactive profile was performed using the BIOPEP-UWM database (Minkiewicz et al., 2019). Absorption, Distribution, Metabolism, Excretion, and Transport (ADMET) for the selected peptides was predicted using the AdmetSAR server. Brain blood barrier (BBB), human intestinal absorption (HIA), *Salmonella typhimurium* reverse mutation assay (AMES) toxicity, carcinogenic potential, acute oral toxicity, and hepatotoxicity were predicted with the help of the AdmetSAR tool (http://lmmd.ecust.edu.cn/admetsar2/).

### Statistical Analysis

Germination and digestion time course experiments were carried out three times. Experimental data represent the mean and SD of the three replicates (*n* = 3). One-way ANOVA using Duncan's test was employed to compare the mean values and identify statistical differences (*p* ≤ 0.05) using Statgraphics Centurion XVIII (Statgraphics Technologies, The Plains, VA, USA). Correlations between traits were analyzed by a Pearson correlation coefficient (*r*). Principal component analysis (PCA) was performed to evaluate associations among germination time, legume type, anti-nutrient content, and proteolysis using Statistica 8.0 (Statsoft, Inc, Tulsa, OK, USA).

## Results and Discussion

### Imbibition, Germination, and Sprout Development Are Faster in Dehulled Lentil

There were remarkable variations in the duration of imbibition among legume seeds. Clearly, DL showed the shortest imbibition time (1 h, Supplementary Figure 1C). The reason for this observation might be an increased water permeability due to the absence of a seed coat that caused a rapid swelling of the seed. The duration of the imbibition phase in GZL, BL, and ZF was 3-, 2-, and 4-fold longer than that found in DL probably due to the presence of a seed coat (Supplementary Figures 1A–D). Additionally, the duration of soaking time in the group of legumes with a seed coat was positively correlated to seed weight (*p* ≤ 0.05). Further germination trials were performed using 3, 2, 1, and 4 h as the optimum soaking times for GZL, BL, DL, and ZF.

Radicle protrusion indicates the end of germination in “*sensu stricto*” that involves the activation of the endogenous metabolism necessary for mobilizing reserve material and radicle growth (Nonogaki et al., 2010). From a visual inspection, it was observed that radicle protrusion occurred after 24 h for GZL, BL, and ZF (Supplementary Figures 2A–D), in contrast the germination of DL occurred faster (15 h, Supplementary Figure 2C). These results indicated that post-germination phase, in which the mobilization of seed storage reserves is mainly mobilized, initiates earlier in DL compared to the other seed types. In consistency with the faster imbibition and germination, the development of DL sprouts occurred earlier (between 24 and 89 h from the end of imbibition) (Supplementary Figure 3C) compared to GZL, BL, and ZF in which a rapid sprout development occurred between 72 and 144 h (Supplementary Figures 3A–D). These results indicated that seed dehulling speeds up the germination and post-germination events. Opposite to DL, ZF showed the slowest sprout development probably associated to its lower rate of water uptake determined by a bigger size of the seed and a reduced surface area.

There is limited mobilization of reserves during germination compared to reserve utilization during post-germination (Nonogaki et al., 2010). Based on this information as an indicative of the period of major mobilization of the reserve material, we selected 96 and 144 h as sampling points to study the nutritional changes on seed proteins in terms of the digestibility, amino acid composition, and bioaccessibility of amino acids and peptides with potential health implications.

### Germination Progressively Reduced Anti-nutrient Content in Lentil and Fava Bean Seed Types

The comparison among lentil types before germination showed that GZL and BL were characterized by higher amounts of TIA values (≈11 TIU/mg) but by a lower phytic acid content (0.8 and 0.9 mg/g, respectively) (Table 1). In contrast, DL showed a significantly (*p* ≤ 0.05) lower amount of TIA (10.2 TIU/mg) but higher phytic acid concentrations (1.1 mg/g) than GZL and BL. Condensed tannins were absent in GZL (zero-tannin variety) and DL in which the removal of a seed coat was the main determinant to this feature (Mirali et al., 2016), whereas BL showed values (4.3 mg/g) comparable to the literature data for tannin content in lentil seeds (Irakli et al., 2021). In comparison with lentil seeds, ZF was characterized by higher TIA (16.1 TIU/mg) and phytic acid (2.6 mg/g) values and 4-fold less concentration of condensed tannins than BL.

**Table 1:** Anti-nutrient and total protein contents in dry seeds (0 h) and sprouts of lentil and fava bean at different times of germination (96 and 144 h).

**Legume type**	**GT (h)**	**TIA** **(TIU/mg)**	**Phytic acid** **(mg/g)**	**Condensed tannins** **(mg/g)**	**Protein** **(g/100 g)**
GZL	0	11.41 ± 0.34^a,B^	0.75 ± 0.03^a,D^	0	27.16 ± 0.15^c,B^
	96	9.09 ± 0.35^c,C^	0.60 ± 0.01^b,C^	0	29.27 ± 0.59^b,A^
	144	9.71 ± 0.11^b,B^	0.47 ± 0.01^c,C^	0	30.13 ± 0.19^a,A^
BL	0	10.96 ± 0.78^a,B^	0.93 ± 0.01^a,C^	4.27 ± 0.12^a,A^	27.57 ± 0.09^b,A^
	96	9.42 ± 0.39^b,C^	0.84 ± 0.02^b,B^	4.43 ± 0.17^a,A^	29.94 ± 0.24^a,A^
	144	9.42 ± 0.55^b,B^	0.71 ± 0.02^c,B^	3.95 ± 0.13^b,A^	30.11 ± 0.45^a,A^
DL	0	10.17 ± 0.79^b,C^	1.12 ± 0.02^a,B^	0	24.51 ± 0.08^c,D^
	96	11.28 ± 0.37^a,B^	0.47 ± 0.02^b,D^	0	27.50 ± 0.17^b,B^
	144	11.20 ± 0.69^a,A^	0.40 ± 0.01^c,D^	0	28.07 ± 0.21^a,B^
ZF	0	16.14 ± 0.47^a,A^	2.59 ± 0.04^a,A^	0.98 ± 0.04^b,B^	24.74 ± 0.04^c,C^
	96	11.91 ± 0.36^b,A^	1.17 ± 0.03^b,A^	1.37 ± 0.03^a,B^	25.76 ± 0.49^b,C^
	144	10.03 ± 0.24^c,B^	1.10 ± 0.04^c,A^	1.40 ± 0.06^a,B^	26.53 ± 0.40^a,C^

*Data are the mean ± SD of the three replicates*.

*Different lowercase letters within a column indicate statistical differences among different germination endpoints (p ≤ 0.05 post-hoc Duncan's test)*.

*Different uppercase letters denote statistical differences among legumes at the same germination time (p ≤ 0.05 post-hoc Duncan's test)*.

*BL, beluga lentil; DL, dehulled red lentil; GT, germination time; GZL, gray zero-tannin lentil; TIA, trypsin inhibitory activity; TIU, trypsin inhibitory units; ZF, low tannin/zero vicine–convicine fava bean*.

There was a general decreasing trend in the amount of anti-nutrients after germination in all legumes studied in agreement with existing evidence (Ohanenye et al., 2020). This effect is related to the activation of dormant enzymes, a physiological response that is influenced by germination conditions, sprouting time, and the type of legume (Donkor et al., 2012). Overall, TIA was significantly (*p* ≤ 0.05) decreased after 96 h of germination by 14.0–26.2% in most legumes' studies from which ZF showed the highest reduction. An exception to this observation was DL in which TIA levels were slightly increased by 11%. The extension of germination time from 96 to 144 h did not cause additional reductions of this activity in most of the seeds, except for ZF that showed a further decrease in TIA up to 35%. The partial degradation of trypsin inhibitors during germination as a result of endogenous protease activation has been recently reviewed (Ohanenye et al., 2020). For instance, cowpea, fava bean, Indian bean, lentil, mung bean, and soybean germinated for 2–5 days decreased TIA by 21.9–23.5, 64.0–65.0, 51.0–83.4, 7.0–45.0, 53.6, and 24.0–29.3%, respectively.

Phytic acid content was reduced time-dependently during germination in all the four legumes studied although the extent of degradation was dependent on the legume type and sprouting time. Specifically, phytic acid content was reduced by 10.2–57.8 and by 23.9–63.8% after 96 and 144 h of germination, respectively. The highest decrease in phytic acid was noticed in DL in which sprouts reached the lowest amount of this anti-nutrient (0.4 mg/g). The increase in endogenous phytase activity has been recognized as the primary cause of phytate degradation during the germination and improvement of mineral, protein, and starch bioaccessibility in germinated cereals, pseudocereals, and legumes (Montemurro et al., 2019). Relatively long sprouting times (3–5 days) are needed to lower the phytate concentration by more than 30% (Ohanenye et al., 2020).

Condensed tannins were significantly (*p* ≤ 0.05) reduced in BL (7.6%) only when germination was extended up to 144 h. An opposite trend was observed for ZF in which the concentration of condensed tannins was 1.4-fold higher regardless of germination time. Previous studies found a reduction in tannin content of common bean, fava bean, Indian bean, peanut, and soybean subjected to germination (Ohanenye et al., 2020) due to the fact that tannins may leach into liquid medium during the soaking phase or can be enzymatically degraded (Chinma et al., 2021).

### Germination Increased Progressively Protein Content in Lentil and Fava Bean Seed Types

Table 1 shows the protein content of dry seeds and sprouted legumes at different times of germination (96 and 144 h). The protein content in lentil types ranged from 24.5 to 27.6% in line with the literature data (Subedi et al., 2021) with the highest values observed for GZL and BL and the lowest for DL (*p* ≤ 0.05). ZF showed a protein content (24.7%) comparable to lentil seeds, which was in agreement with previous studies (Warsame et al., 2018). Germination brought about a gradual and significant time-dependent increase in the protein content in lentil and fava bean types (except BL in which there was no significant (*p* > 0.05) difference between 96 and 144 h). This effect was more pronounced in DL with a 12.2% and 14.6% higher protein content after 96 and 144 h of germination, respectively. These results are in accordance with a previous study reporting that germination slightly increased the protein content of chickpea, lentil, and yellow pea by 3.4, 2.9, and 2.8%, respectively (Xu et al., 2019). The observed effect could be explained by the *de novo* synthesis of proteins during germination or a seed compositional change following the degradation of other constituents such as carbohydrates through respiration (Ohanenye et al., 2020).

### Germination Slightly Reduced EAA to Non-essential Amino Acids Ratio in Lentil and Fava Bean Seed Types

The distribution of EAA and non-essential amino acids (NEAA) in dry seeds and sprouted legumes at different times of germination (96 and 144 h) is shown in Table 2. E, D, and R were found to be the major NEAA in lentil and fava bean seeds, whereas sulfur amino acids C and M were limiting compared with the Joint FAO/WHO Ad Hoc Expert Committee on Energy and Protein Requirements, World Health Organization, and Food and Agriculture Organization of the United Nations (1973) reference pattern. These results are in agreement with the values reported by several authors (Khazaei et al., 2019; Warsame et al., 2020). EAA/NEAA ratio was similar between GZL and DL (45%:55% = 0.81) and higher than the ratio calculated for BL and ZF (39%:61% = 0.64 and 38%:61% = 0.62, respectively). In general, this parameter was minimally reduced in GZL, DL, and ZF after 96 h of germination (EAA/NEAA ratio: 0.76, 0.77, and 0.58, respectively), except for BL in which the distribution of amino acids was not significantly (*p* > 0.05) modified. This decreasing trend observed for the EAA/NEAA ratio reached the lowest values after 144 h in GZL, DL, and ZF (0.67, 0.69, and 0.56, respectively).

**Table 2:** The distribution of essential amino acids (EAA) and non-essential amino acids (NEAA) in raw and sprouted legume flours at different times of germination (96 and 144 h).

	**AA**	**GT (h)**	**% of total AA**			
			**GZL**	**BL**	**DL**	**ZF**
EAA	Arg	0	6.32	5.25	5.91	5.85
		96	5.35	4.81	4.92	5.13
		144	4.91	4.57	4.47	4.97
	Thr	0	4.20	3.21	4.14	2.56
		96	4.15	3.60	3.99	2.59
		144	3.87	3.61	3.80	2.55
	His	0	2.92	2.76	2.87	2.74
		96	2.92	2.87	2.82	3.17
		144	2.95	2.97	2.74	3.27
	Lys	0	8.86	8.62	8.24	8.10
		96	9.07	8.56	7.89	8.23
		144	7.85	8.68	7.55	7.95
	Phe	0	5.17	5.99	5.31	6.86
		96	5.15	5.71	5.54	6.87
		144	5.13	5.42	5.26	6.55
	Leu	0	7.60	6.37	7.98	5.83
		96	7.13	6.53	7.98	5.77
		144	6.57	6.31	7.69	5.67
	Met	0	0.62	0.31	0.63	0.37
		96	0.58	0.39	0.66	0.34
		144	0.63	0.33	0.55	0.33
	Ile	0	4.07	2.62	4.28	2.62
		96	3.71	2.85	4.41	2.67
		144	3.35	2.77	3.94	2.52
	Val	0	5.12	3.90	5.26	3.37
		96	5.05	4.18	5.41	4.01
		144	4.82	4.21	5.00	4.20
NEAA	Cys	0	0.55	0.78	0.65	0.93
		96	0.65	0.59	0.60	0.93
		144	0.71	0.41	0.50	0.92
	Tyr	0	2.37	1.78	2.51	3.33
		96	2.19	1.52	2.51	3.26
		144	2.39	1.32	2.14	3.07
	Pro	0	4.43	4.51	4.10	3.93
		96	4.51	4.81	4.32	4.54
		144	5.06	4.99	4.63	4.88
	Ala	0	4.99	5.85	5.09	6.03
		96	4.98	5.35	4.82	6.81
		144	5.00	5.13	5.03	6.58
	Gly	0	4.93	6.21	5.03	7.05
		96	4.56	4.96	4.48	6.88
		144	4.45	4.82	4.61	6.65
	Ser	0	6.30	7.15	6.16	6.12
		96	6.58	7.15	6.10	6.97
		144	6.53	7.05	5.90	7.11
	Glu+Gln	0	18.17	20.40	18.82	20.87
		96	16.48	18.14	16.47	21.48
		144	15.89	17.02	17.00	20.83
	Asp + Asn	0	13.40	14.31	13.03	13.43
		96	16.95	17.99	17.08	15.52
		144	19.90	20.40	19.18	17.94

*Data are the mean of the three replicates*.

*AA, amino acids; Ala, alanine; Arg, arginine; Asn, asparagine; Asp, aspartic acid; BL, beluga lentil; Cys, cysteine; DL, dehulled red lentil; Gln, glutamine; Glu, glutamic acid; Gly, glycine; GT, germination time; GZL, gray zero-tannin lentil; His, histidine; Ile, isoleucine; Leu, leucine; Lys, lysine; Met, methionine; Phe, phenylalanine; Pro, proline; Ser, serine; Thr, threonine; Tyr, tyrosine; Val, valine; ZF, low tannin/zero vicine–convicine fava bean*.

### Germination Increased Protease Activity and Activated Proteolysis of Storage Proteins in Lentil and Fava Bean Seed Types

**Figure 1:**
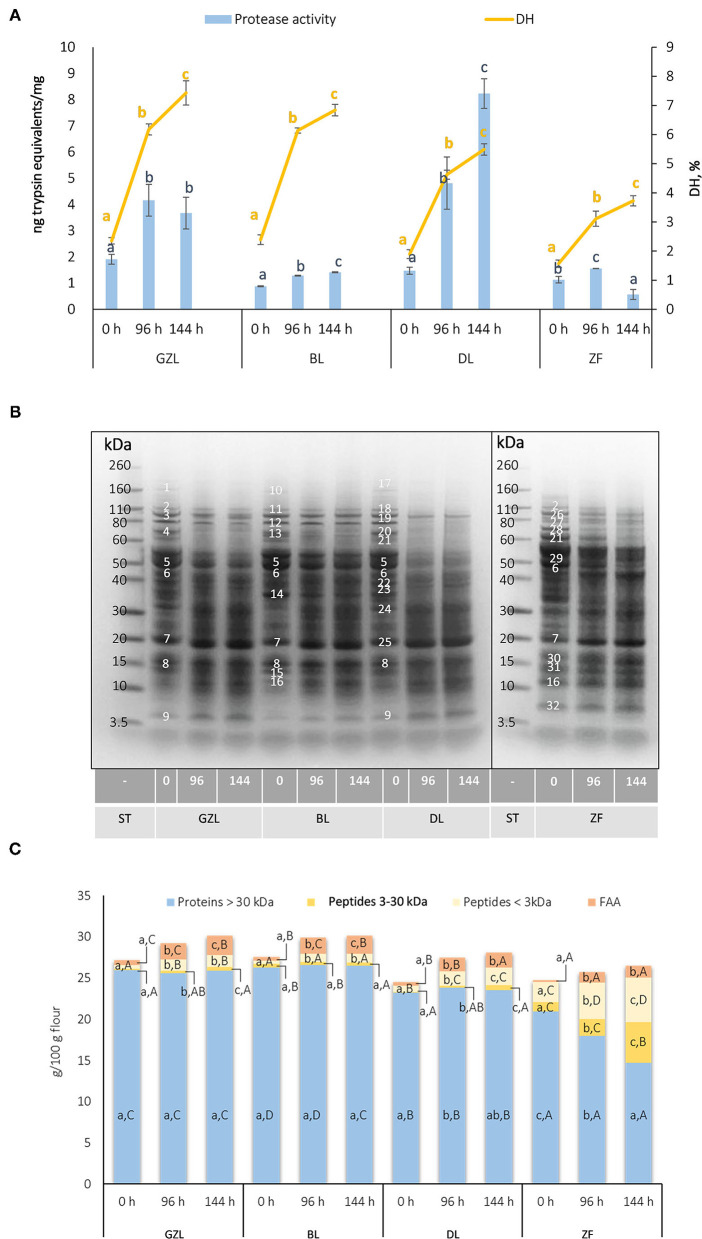
**(A)** Protease activity and the degree of protein hydrolysis (DH) in ungerminated (0 h) and sprouted lentil and fava bean seeds at different germination times (96 and 144 h). Data are the mean ± SD of the three replicates. Different uppercase letters denote statistical differences among legumes at the same germination time (*p* ≤ 0.05 *post-hoc* Duncan's test). **(B)** A pattern of proteolysis in ungerminated seeds (0 h) and sprouts of lentil and fava bean at different germination times (96 and 144 h). Representative Coomasie-blue stained gels. 1: Unknown protein, 164 kDa; 2: Lipoxygenase isoform 1/Linoleate-9s-lipoxygenase-3 isoform 1, 111 kDa; 3: Lipoxygenase isoform 2, 93 kDa; 4: Convicilin, 68 kDa; 5: Vicilin, 53 kDa; 6: Vicilin, 48 kDa; 7: Vicilin precursor/Legumin β, 20kDa; 8: 2S albumin, 15 kDa; 9: 2S albumin, 4 kDa; 10: Unknown protein, 115 kDa; 11: Lipoxygenase isoform 1, 107 kDa; 12: Lipoxygenase isoform 2, 90 kDa; 13: Convicilin, 66 kDa; 14: Legumin, 35 kDa; 15: 2S albumin, 13 kDa; 16: 2S albumin/Albumin 1, 11kDa; 17: Unknown protein, 170 kDa; 18: Lipoxygenase isoform 1, 109 kDa; 19: Lipoxygenase isoform 2, 91 kDa; 20: Convicilin, 67 kDa; 21: Convicilin, 61 kDa; 22: Legumin, 42 kDa; 23: Legumin, 39 kDa; 24: Legumin, 31 kDa; 25: Vicilin precursor, 19 kDa; 26: Linoleate-9s-lipoxygenase-3 isoform 2, 94 kDa; 27: Convicilin, 79 kDa; 28: Heat shock protein, 70 kDa; 29: Convicilin, 54 kDa; 30: Legumin β, 16 kDa; 31: Albumin 1, 14 kDa; 32: Unknown peptide, 6 kDa; ST, Molecular weight marker. **(C)** The distribution of protein content between proteins >30 kDa, peptides 3–30 kDa, peptides <3 kDa, and FAA in dry seeds and sprouted lentil and fava bean at different times of germination (96 and 144 h). Data are the mean ± SD of the three replicates. Different lowercase letters indicate statistical differences among different germination endpoints (*p* ≤ 0.05 *post-hoc* Duncan's test). Different uppercase letters denote statistical differences among legumes at the same germination time (*p* ≤ 0.05 *post-hoc* Duncan's test). FAA, free amino acids; GZL, gray zero-tannin lentil; BL, beluga lentil; DL, dehulled red lentil; ST, molecular weight standard; ZF, low tannin/zero vicine–convicine fava bean.

Protease activity in dry seeds varied from 0.9 to 1.9 ng trypsin equivalents/mg flour, with the highest values observed for GZL (Figure 1A, blue bars). A gradual increase in the protease activity over germination time was observed in most of the cases studied, except for ZF that showed a drop of this parameter by 63% from 96 to 144 h. The highest increase in protease activity during germination was observed for DL (3.3- and 5.6-fold after 96 and 144 h, respectively). Our results are in agreement with previous studies showing a 2- to 3-fold increase in protease activity in soybean when germinated for 2–6 days at 25°C (Zou and Hou, 2017). Maximum values were reached at longer germination times (5 and 6 days). Germination is associated with the activation of endogenous papain- and legumain-type cysteine proteases that are essential for seedling development as they degrade storage proteins to release FAAs for *de novo* synthesis of proteins (Ohanenye et al., 2020).

The proteolysis of the four studied legume types occurred after germination for 96 and 144 h was monitored by the analysis of protein degradation by the DH (Figure 1A, yellow lines), by SDS-PAGE (Figure 1B), and the release of peptides of different MW and FAAs (Figure 1C). The results showed that there was a time-dependent release of free amino groups in the four legumes studied, in consistency with an increase in protease activity during germination. A clear variation was observed in the kinetic behavior of protein hydrolysis among species. Lentil samples (GZL, BL, and DL) showed a higher release of free amino groups compared to ZF that showed a lower DH at each germination time point. These results could be associated with contrasting imbibition rates and speed of sprout development (Supplementary Figures 1, 3) between lentil and fava bean.

As shown in Figure 1B, the three lentil types had a typical electrophoretic profile including polypeptides corresponding to 2S albumins (4–15 kDa), vicilin precursor (20 kDa), legumins (31–42 kDa), vicilins (49 and 53 kDa), convicilins (60–70 kDa), and lipoxygenase isoforms (90–110 kDa) according to the literature (Scippa et al., 2010; Ialicicco et al., 2012). Fava bean seeds showed 14 major protein bands in the MW range from 6 to 111 kDa. The most abundant proteins in ZF were unknown peptide (6 kDa), albumin-1 polypeptides (11 and 14 kDa), polypeptides belonging to legumin β (16, 20, and 43 kDa), vicilin (30 and 48 kDa), and convicilin (54, 61, and 79 kDa), heat shock protein (70 kDa), and linoleate 9S-lipoxygenase-3 isoforms (111 and 94 kDa) (Warsame et al., 2020).

Germination decreased the optical density of major storage protein bands (vicilins, convicilins, and legumins) concurrently with the increased intensity of low MW bands in all lentil types studied, being these effects more pronounced at 144 h of germination (Figure 1A). Densitometric analysis of protein bands indicated that after germination vicilin, convicilin, legumin, and lipoxygenase polypeptides are reduced between 11–25, 1–21, 11–41, and 2–21% in GZL, BL, DL, and ZF, respectively (Supplementary Figure 4). Moreover, 7S globulins were earlier and greater degraded than 11S globulins during germination. At the same time, intensities of bands with MW between 4–30 kDa increased by 7–22, 7–31, 8–33, and 2–19% in GZL, BL, DL, and ZF, respectively. In consistency with protease activity, proteolysis was more evident in DL, whereas it was less pronounced in ZF. Therefore, the extent of protein degradation and the generation of new protein fragments with lower MW seem to be linked to seed permeability, the speed of imbibition, germination, and post-germination (Zahir et al., 2020). In consistency with our results, several studies have shown that the degradation of legumin starts later during germination, after vicilin; as such, legumin represents the bulk reserve source for amino acids in the legume seeds. For instance, electrophoretic profiles of yellow pea proteins showed the disappearance of 7S globulins (convicilin and vicilin) earlier in the germination than 11S globulins (legumin) (Di Stefano et al., 2019). Similarly, β-conglycinin was degraded earlier than the acidic glycinin subunit while the basic chain was more resistant (Kim et al., 2011).

The distribution of the nitrogen fraction in dry seeds and sprouted legumes at different times of germination is shown in Figure 1C. FAAs, peptides <3 kDa, and polypeptides from 3 to 30 kDa represent a small portion of the total nitrogen fraction (1.1–2.3, 1.6–10.7, and 0.7–4.9%, respectively) in the four dry seed types. ZF showed a lower content of FAA but the highest amounts of peptides with MW <3 kDa and from 3 to 30 kDa compared to lentil seeds, differences that could be associated to species-specific profiles of the nitrogen fraction. After germination, an increase of low molecular protein fragments and FAAs was observed in all the samples studied. There was a time-dependent increase of protein fragments with MW from 3 to 30 kDa and below 3 kDa in DL and ZF, whereas FAAs increased gradually up to 144 h of germination only in BL. The highest increase of FAAs caused by germination was observed for ZF (5- to 6-fold change after 96 and 144 h vs. dry seeds). On the other hand, GZL and DL sprouts showed the highest increases (almost a 3-fold change regardless of time) in the concentration of peptides <3 kDa. In terms of absolute concentration, GZL sprouts had the highest FAA content (2.0 and 2.3 g/100 g flour after 96 and 144 h of germination, respectively), whereas ZF sprouts were characterized for the highest levels of peptides with MW <3 kDa (4.4 and 5.3 g/100 g flour after 96 and 144 h of germination, respectively) and from 3 to 30 kDa (2.1 and 5.0 g/100 g flour after 96 and 144 h of germination, respectively).

### Germination-Induced Changes on Protein Fraction and Anti-nutrient Content Varied With Time and Among Legume Type

Principal component analysis was applied to explore a variation in the effect of germination on the protein content, anti-nutrient content, and markers of proteolysis (DH, FAAs, peptides with MW <3 kDa and between 3 and 30 kDa) as influenced by post-germination time and legume type factors. The first two components accounted for 63.71% of the total variance (Supplementary Figure 5). The first component (factor 1) explained 44.09% of the variance and had positive loadings for TIA and phytic acid, whereas it was negatively influenced by the total protein content, FAAs, DH, and germination time (Supplementary Figure 5A). The second component (factor 2, 19.62% of the variability) had positive loadings for peptide content (<3 kDa and between 3 and 30 kDa). Furthermore, the protein content and markers of proteolysis such as FAAs and DH were positively correlated with germination time (*r* = 0.61, 0.91, 0.82, *p* ≤ 0.05). Furthermore, DH was positively correlated with FAAs and negatively correlated with TIA and phytic acid content (*r* = 0.97, −0.63, −0.64, *p* ≤ 0.05).

The score plot of the two principal components (Supplementary Figure 5B) showed a high variation in the extent of proteolysis and anti-nutrient content between legumes and germination time. The proximity among cases reflected similarities in composition. When analyzing this plot for the discrete variable “germination time”, it was clear that the great differences between dry and germinated seeds although extending the germination time from 96 to 144 h did not result in greater changes in the composition of sprouts with the exception of ZF. Dry seeds clustered on the right side (positive) of a bi-plot, indicating their higher content in TIA and phytic acid and lower protein content and markers of proteolysis than sprouts regardless of sprouting time; however, germination changes differed significantly among seed types. ZF sprouts decreased in principal components 1 and 2, indicative of a slower rate of proteolysis based on a progressive increase in the protein content, a degradation of anti-nutrients, and an increase of protein breakdown products with higher MWs (peptides < 3 kDa and between 3 and 30 kDa) up to 144 h of germination. These changes were also observed in the case of lentil types although they were located on the left side of the score plot, due to a greater protein content, a higher inactivation of anti-nutrients, and the accumulation of the proteolysis end products (FAAs) compared to ZF sprouts. GZL and BL experienced similar nutritional changes after germination in terms of anti-nutrients and protein mobilization, as confirmed by their proximity in the score plot regardless of sprouting time; however, DL was clustered with different coordinates due to its lower anti-nutrient content and more extensive proteolysis.

Due to the similarities found in legumes germinated for 96 and 144 h in most of the seed types included in the current study, the shorter germination time was selected as an representative to observe a variation in the effect of germination on seed microstructure, protein digestibility, and bioactivity across seed types.

### Germination Degrades Cellular Structure in a Variable Extent Depending on the Legume Seed Type

As mentioned earlier, germination triggers partial digestion of seed proteins, which can be translated into higher digestibility and nutritional value when consumed by humans. Nonetheless, an intact cellular structure has been identified as a critical factor affecting the rate and extent of seed protein digestion (Becker and Yu, 2013). A thick and compact seed coat and cell walls (CWs) of cotyledon represent the physical barriers that limit the activity of digestive enzymes although germination may improve amino acid bioavailability through modifications on the integrity of plant CWs and the dissociation of nutrient–matrix complexes (Zahir et al., 2020). Therefore, changes in the seed microstructure of lentil and fava bean types as affected by germination for 96 h were investigated.

**Figure 2:**
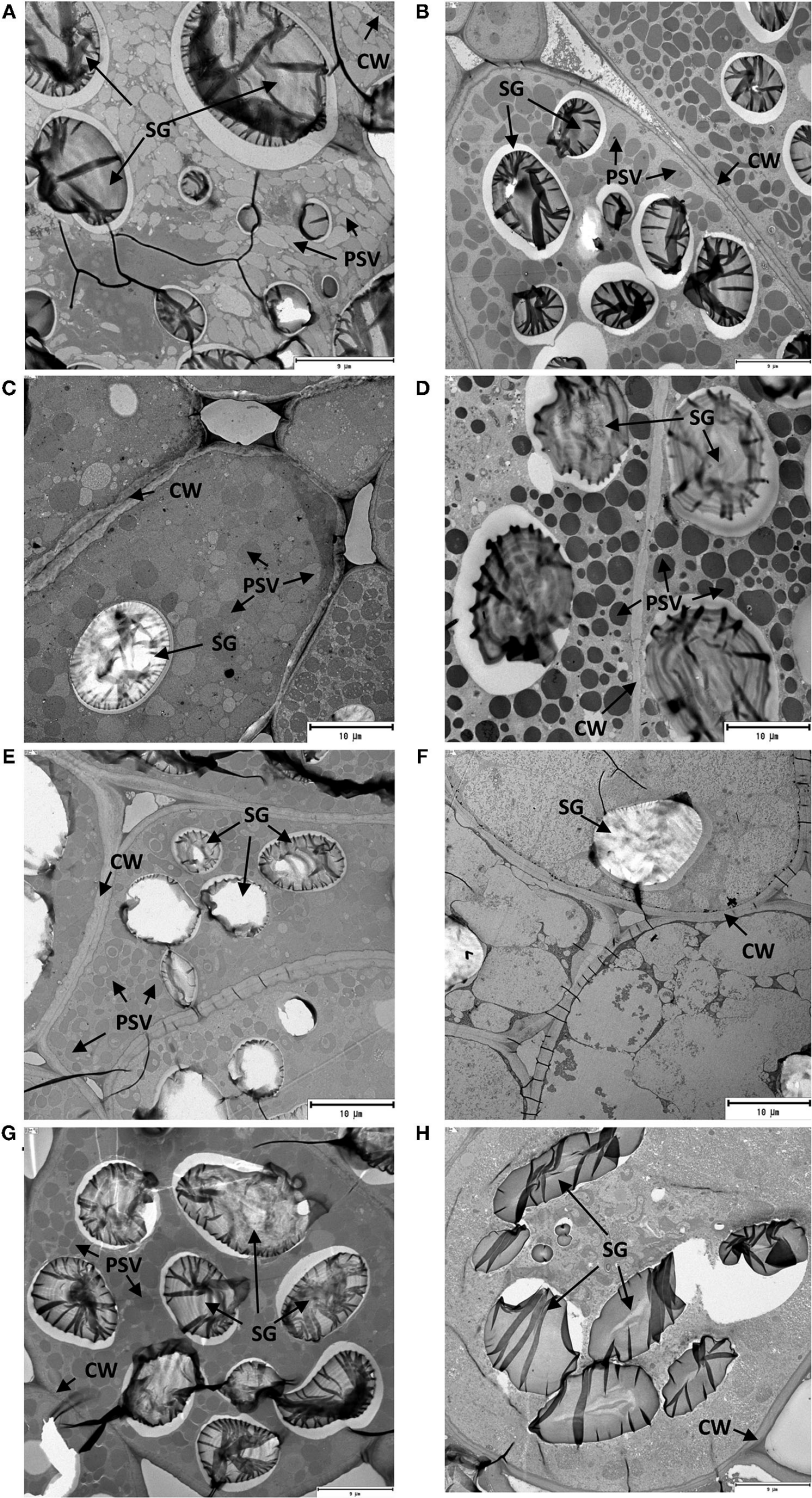
Transmission electron micrographs of cotyledons from the soaked (**A,C,E,G** corresponding to GZL, BL, DL, and ZF, respectively) and sprouted lentil and fava bean seeds at 20°C for 96 h (**B,D,F,H** corresponding to GZL, BL, DL, and ZF, respectively).

Changes observed in the cellular structure of parenchyma cells in the mid-region of cotyledons of the soaked and germinated legume seeds are shown in Figure 2. The two main nutrient reserves of legume seeds, protein and starch, were stored in the specialized organelles of seed cotyledon parenchyma cells such as protein storage vacuoles (PSV) and starch granules (SG), respectively. An examination of the parenchyma cells after soaking of GZL, BL, DL, and ZF seeds (Figures 2A–G, respectively) revealed a compact cellular structure covered by an intact CW that was filled with numerous PSV of various sizes and large-size SG. Other organelles, including rough endoplasmic reticulum and Golgi apparatus, were not observed in these cells. The observation of parenchyma cells of sprouted legumes at 20°C for 96 h (Figures 2B–H) presented a different anatomy compared with cotyledon images of non-germinated seeds. It could be clearly seen that cotyledons of sprouted seeds showed a lower density of PSV with heterogeneous sizes. In a particular case of DL, the depletion of PSV and emptying of these organelles were more evident (Figure 2F), suggesting a more extensive mobilization of storage proteins as compared to GZL, BL, and ZF. This observation was consistent with shorter imbibition, germination, and post-germination phases observed for DL (Supplementary Figures 1, 3) that resulted in higher protease activity and protein degradation (Figures 1A,B) in comparison with other seed types. By comparing the micrographs of the other seed types having a seed coat, minor changes were observed for BL and ZF after 96 h from the initiation of germination. These results were, indicative of delayed post-germination changes in seed microstructure compared to GZL that could be attributed to low seed permeability for BL and a bigger seed size in the case of ZF. Similarly, a study on five legumes (fava bean, yellow field pea, green lentil, chickpea, and kidney bean) demonstrated that microstructure changes induced by germination did not follow the same degradation pattern in all legume types (Di Stefano et al., 2019). The germination of yellow field pea led to parenchyma cell disappearance, an increase in CW thickness and a depletion in SG protein bodies, whereas in green lentil both the seed coat and CW appeared thin after germination.

Based on existing evidence, changes in the cellular microstructure observed in our study suggest that germination may have initiated the permeabilization of CWs and the predigestion of storage proteins attached to cell structure (Table 1). This hypothesis is supported by the study of Zahir et al. (2020) who demonstrated that partial degradation/solubilization of pectin and other polymers of the CW in germinated soybean seeds led to extensive cellular permeability to dextrans of high molecular sizes (up to 150 kDa). It is important to highlight that, in agreement with our results, the extent of these changes varies among legume types. For instance, a higher microstructure degradation and consequently the degree of proteolysis were reported for green lentil, yellow pea, chickpea, and fava bean after 5 days of germination compared to a kidney bean, which showed the lowest protein bioaccessibility (Di Stefano et al., 2019).

### Germination Favored Protein Digestion in Lentil and Fava Bean Seed Types

**Figure 3:**
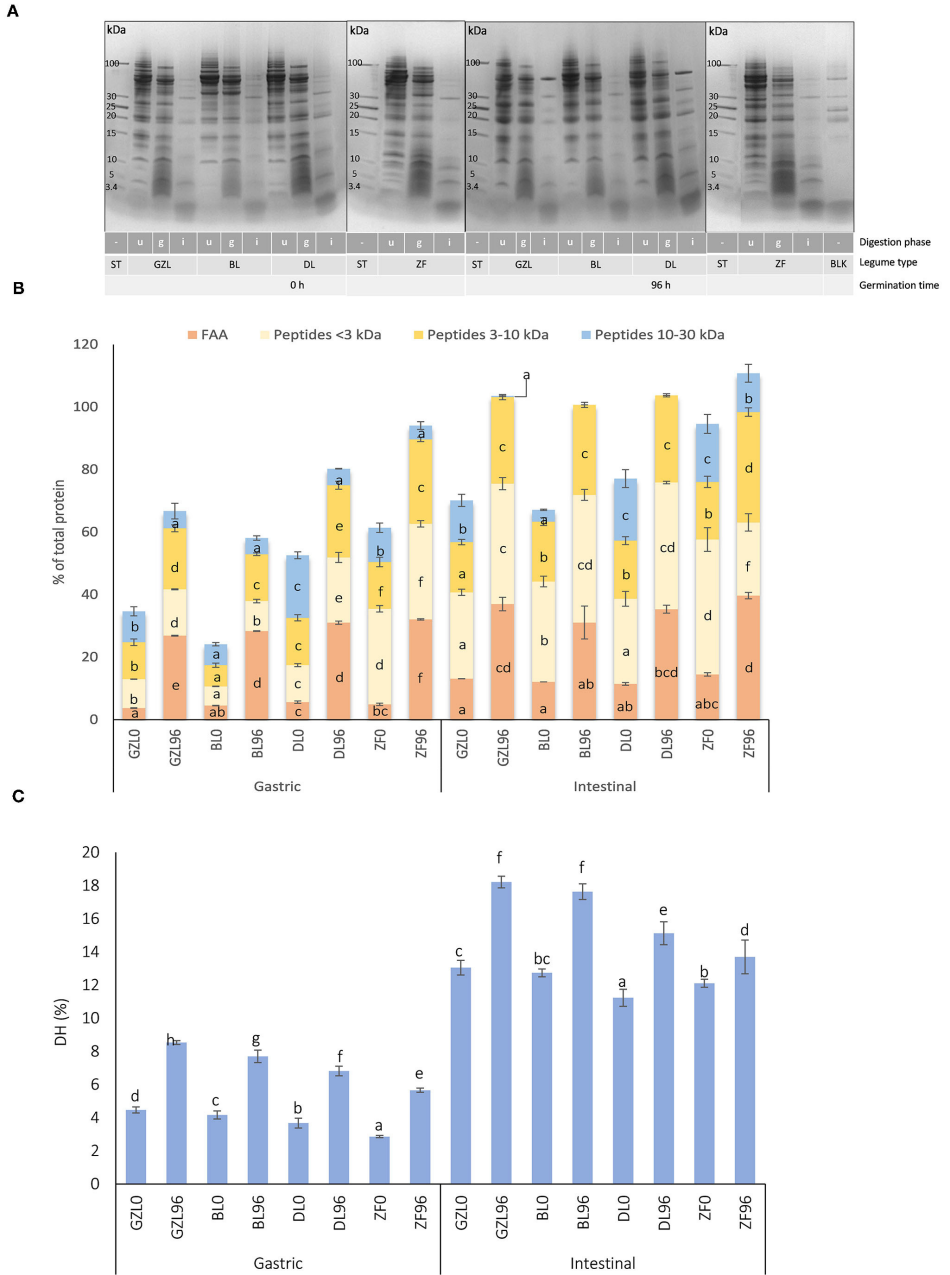
**(A)** SDS-PAGE protein profiles of dry and sprouted lentil and fava bean seeds (20°C, 96 h) at different times of *in vitro* gastrointestinal digestion. **(B)** The distribution of nitrogen fraction after the simulated gastric and intestinal digestion of lentil and fava bean seed types before (GZL0, BL0, DL0, and ZF0) and after seed sprouting at 20°C for 96 h (GZL96, BL96, DL96, and ZF96). **(C)** Degree of hydrolysis (DH) after the simulated gastric and intestinal digestion of dry and sprouted lentil and fava bean seeds at 20°C for 96 h. Data and error bars represent the mean and SD, respectively, of the three replicates. In each analysis performed, different letters indicate statistical differences among legumes at the same digestion phase (*p* ≤ 0.05 *post-hoc* Duncan test). BL, beluga lentil; BLK, blank of digestion (contains all reagents and enzymes used in digestion; DL, dehulled red lentil; ZF, low tannin/zero vicine–convicine fava bean; GZL, gray zero-tannin lentil; g, end of gastric digestion (120 min); i, end of digestion in the small intestine (240 min); ST: molecular weight marker; u, undigested sample.

Digestion kinetics of proteins in dry and sprouted seeds was followed by SDS-PAGE. A representative gel is illustrated in Figure 3A where a gradual degradation of the main storage proteins (vicilin, convicilins, and legumins) and the generation of smaller breakdown products in dry and sprouted seeds can be seen. Regardless of sample treatment, vicilin- and legumin-type globulins were partially resistant to pepsin digestion in lentil and fava bean types. Clear differences in the protein profile of lentil and fava bean were observed between dry and sprouted seeds at the end of the gastric phase (g lanes). A lower intensity of bands corresponding to convicilins, vicilins, and legumins and a higher intensity of peptide bands of MW <10 kDa were noticed in sprouted samples as compared to dry seeds (u lanes). These results suggested a more rapid digestion of proteins by pepsin in GZL, BL, DL, and ZF sprouted seeds. As it is well known that the hydrolysis of proteins by pepsin is very limited (Santos-Hernández et al., 2020), the higher protein hydrolysis in the gastric phase demonstrates the role of the germination process in “pre-digesting” proteins into smaller peptides and, eventually, leading to more digestible proteins in the gastric phase.

At 120 min of intestinal digestion (i lanes), most of the protein bands had fully disappeared and only those with MW under 15 kDa became more intense, which probably correspond to incomplete protein degradation products. Furthermore, new bands having MWs ~24, 36, 38, and 52 kDa, also visible in the blank of digestion (blank of digestion (BLK) lane), might belong to digestive enzymes pepsin (36 kDa) and pancreatin (23–27, 38, and 51–54 kDa) including trypsin, amylase, lipase, ribonuclease, and protease (Santos-Hernández et al., 2020). Similar to the results found in the gastric phase, visible differences were observed between intestinal digesta of dry and sprouted seeds in which higher intensities in bands with MWs <15 kDa could be seen. Similar observations were described by Santos-Hernández et al. (2020) and Sousa et al. (2020) who reported that the partial resistance of some legume proteins such as vicilin, provicilin, convicilin, lipoxygenase, legumin A, and legumin A2 during the gastric phase and an almost complete degradation at the end of the intestinal phase joined to the appearance of new electrophoretic bands below 15 kDa.

The evolution of MW distribution of the nitrogen fraction up to 30 kDa in dry and sprouted seeds during digestion is shown in Figure 3B. Compared with the intestinal phase, nitrogen fraction of gastric digesta was composed of a lower percentage of FAAs and small peptides independent of the legume type and germination. For instance, after gastric digestion of ungerminated GZL, 3.7, 21.0, and 9.9% of the total nitrogen fraction were comprised of FAAs, peptides (up to 10 kDa), and proteins between 10 and 30 kDa, respectively; and this percentage increased up to 13.1, 43.7, and 13.3%, respectively, at the end of intestinal digestion. A similar response was obtained for BL, DL, and ZF. Higher peptide solubilization and FAA release in the intestinal phase are typically observed with other legume protein isolates from garden pea, grass pea, soybean, and lentil submitted to the INFOGEST method (Santos-Hernández et al., 2020).

In line with peptides and FAA release, DH (Figure 3C) reached the highest levels in the intestinal phase regardless of seed type and germination. By comparing dry and sprouted seeds for 96 h, a significantly higher DH was observed after germination for all the legume types either in gastric and intestinal phases of digestion. Our results are consistent with those reported by Ghavidel and Prakash (2007) who showed that the *in vitro* protein digestibility increased by a range of 14–18% after the germination of green gram, cowpea, lentil, and chickpea.

Because the seed coat and CW are the primary barriers to protein digestibility, a relatively compact intracellular environment may serve as an additional barrier for enzyme permeability and diffusion in intact seed cells (Zahir et al., 2020). Thus, loosely packed parenchyma cells that are observed after sprouting may have favored the permeability and diffusion of digestive enzymes, explaining a greater proteolysis rate observed for lentil and fava bean types in this study together with anti-nutrient reduction, protein solubilization, and predigestion, compared to dry seeds. In addition, a lower content of β-sheet and higher contents of the random coil, α-helix, and β-turn have been reported in germinated legumes as compared to dry seeds (Zahir et al., 2021). The helical structure may expose more peptide bonds of proteins for enzymatic hydrolysis compared to the sheet structure (Martinez-Velasco et al., 2018). Moreover, less folded proteins are more digestible than highly folded proteins (Joye, 2019). Therefore, in this study, protein structural changes that occurred after seed germination may provide additional support to the increased *in vitro* protein digestibility observed for lentil and fava bean sprouts.

By comparing the four sprouted legumes, at the end of the gastric phase, DL and ZF showed higher percentages of FAA (31 and 32%, respectively) and peptides (44 and 57%) than GZL and BL in which the total fraction was made up of 26 and 28% of FAAs and 34 and 24% of peptides up to 10 kDa, respectively. At the end of intestinal digestion, a similar distribution of the nitrogen fraction was found for the three sprouted lentils in which peptides <3 kDa were the most abundant protein breakdown products (38–41% of total nitrogen) followed by FAA (31–36% of total nitrogen) and peptides between 3 and 10 kDa (28–29% of total nitrogen). A different profile was observed for sprouted ZF at the end of intestinal digestion that showed the highest percentage of FAAs (40% of total nitrogen) and peptides in the range 3–10 kDa (35% of total nitrogen), the lowest percentage of peptides <3 kDa (23% of total nitrogen) and a considerable percentage of proteins between 10 and 30 kDa (12% of total nitrogen). In line with these results, sprouted ZF showed the lowest DH (13.7%) as compared to GZL, BL, and DL (18.2, 17.6, and 15.2%, respectively) (Figure 3C), which could be associated with a slow germination process as compared to lentil seed types. By comparing the distribution of nitrogen fraction between dry and sprouted lentil and fava bean types, a positive effect of germination in protein digestibility was evident. Percentages of protein breakdown products in sprouted lentil and fava bean at the end of intestinal phase were higher compared to recently reported values for intestinal digests of garden pea, grass pea, soybean, and lentil (66–76% and 20–30% of soluble nitrogen are peptides and FAAs) (Santos-Hernández et al., 2020).

### Germination Improved Bioactive Traits of Lentil and Fava Bean Types Before and During Digestion

To get additional insights into the antihypertensive and antioxidant properties of peptides generated by germination and subsequent gastrointestinal digestion, their ability to inhibit ACE activity and to scavenge peroxyl (ORAC method) and ABTS radicals (ABTS method) was screened (Table 3). There was a clear variation in the ACE inhibitory activity of dry seeds, of which GZL and ZF showed the highest antihypertensive potential (IC_50_ = 0.50 and 0.63 mg/ml). *In vitro* digestion affected differently this parameter depending on the legume type, digestion phase, and germination. In general, ACE inhibitory activity significantly increased in the gastric phase of digestion (except for the dry seeds of BL, BL0) and was maintained up to the end of intestinal digestion with the exception of GZL and ZF sprouts in which IC_50_ values significantly increased. These results indicate that simulated gastrointestinal digestion of different legume types generates peptides with different sizes, sequences, and structures that potentially explain the differences observed in terms of bioactivity (Dugardin et al., 2020). By comparing dry and sprouted seeds, clear differences were observed at each phase of digestion. A similar antihypertensive potential was noticed between dry and germinated seeds at the end of gastric digestion. However, a higher ACE inhibitory activity was shown for GZL and BL sprouts as compared to dry seeds at the end of intestinal digestion, whereas this parameter remained without any changes for DL or even decreased for ZF.

**Table 3:** ACE inhibitory activity, ORAC, and ABTS scavenging activity of raw and sprouted legumes at different phases of gastrointestinal digestion.

**Legume**	**GT** **(h)**	**Digestion phase**	**ACE inhibitory activity** **(IC_50_, mg/mL)**	**ORAC** **(mmol TE/ g)**	**ABTS** **(mmol TE/ g)**
GZL	0	u	0.50 ± 0.02^cd,F^	35.57 ± 2.93^f,E^	30.05 ± 2.91^e,D^
		g	0.50 ± 0.02^cd,BC^	69.72 ± 6.61^d,F^	67.28 ± 3.77^d,D^
		i	0.63 ± 0.04^b,A^	118.38 ± 10.86^b,C^	97.15 ± 2.55^b,CD^
	96	u	1.01 ± 0.01^a,E^	45.32 ± 3.44^e,D^	31.47 ± 3.06^e,D^
		g	0.44 ± 0.01^d,CD^	89.81 ± 6.77^c,BC^	78.57 ± 6.80^c,C^
		i	0.56 ± 0.01^c,ABC^	150.69 ± 14.62^a,B^	107.11 ± 2.89^a,C^
BL	0	u	0.63 ± 0.02^b,F^	62.67 ± 5.08^c,B^	41.14 ± 4.36^d,C^
		g	0.66 ± 0.04^b,A^	82.04 ± 3.78^b,D^	61.83 ± 5.72^c,E^
		i	0.61 ± 0.00^b,AB^	158.75 ± 11.75^a,B^	88.49 ± 4.51^b,D^
	96	u	4.24 ± 0.07^a,A^	49.79 ± 4.28^d,C^	26.58 ± 2.61^e,E^
		g	0.57 ± 0.00^bc,ABC^	83.14 ± 6.73^b,D^	58.52 ± 5.11^c,EF^
		i	0.47 ± 0.02^c,C^	159.12 ± 12.88^a,B^	101.62 ± 8.80^a,C^
DL	0	u	2.48 ± 0.02^b,C^	34.68 ± 3.37^f,E^	26.82 ± 2.64^c,E^
		g	0.57 ± 0.10^c,ABC^	75.31 ± 6.21^d,E^	54.55 ± 4.74^b,F^
		i	0.51 ± 0.02^c,AB^	127.87 ± 12.16^b,C^	98.60 ± 5.00^a,CD^
	96	u	4.05 ± 0.05^a,B^	45.08 ± 4.49^e,D^	23.19 ± 2.29^c,F^
		g	0.58 ± 0.02^c,AB^	87.49 ± 7.10^c,CD^	58.42 ± 5.16^b,EF^
		i	0.48 ± 0.03^c,C^	154.05 ± 14.98^a,B^	99.00 ± 9.66^a,CD^
ZF	0	u	0.63 ± 0.02^b,F^	60.13 ± 5.30^f,B^	59.15 ± 3.78^f,B^
		g	0.32 ± 0.00^c,D^	94.58 ± 7.49^d,B^	89.21 ± 8.78^d,B^
		i	0.37 ± 0.05^c,D^	151.53 ± 13.30^b,B^	131.99 ± 11.98^b,B^
	96	u	1.29 ± 0.07^a,D^	87.80 ± 7.88^e,A^	78.13 ± 4.48^e,A^
		g	0.44 ± 0.01^c,CD^	128.97 ± 11.92^c,A^	117.01 ± 6.67^c,A^
		i	0.61 ± 0.03^b,AB^	186.13 ± 10.33^a,A^	152.10 ± 14.73^a,A^

*Data are the mean and SD of the three replicates*.

*Different lowercase letters within a column indicate statistical differences among different germination endpoints (p ≤ 0.05 post-hoc Duncan's test)*.

*Different uppercase letters denote statistical differences among legumes at the same germination time (p ≤ 0.05 post-hoc Duncan's test)*.

*ABTS, 2,2′-azino-bis (3-ethylbenzthiazoline-6-sulfonic acid); ACE, angiotensin I converting enzyme; BL, beluga lentil; DL, dehulled red lentil; GZL, gray zero-tannin lentil; g, end of gastric digestion (120 min); GT, germination time; i, end of digestion in the small intestine (240 min); IC_50_, half maximal inhibitory concentration; ORAC, oxygen radical absorbance capacity; TE, Trolox equivalents; u, undigested sample; ZF, low tannin/zero vicine–convicine fava bean*.

Although the generation of ACE inhibitory peptides has been reported after legume sprouting in some studies, legume seed germination may not always lead to the production of ACE inhibitors (Maleki and Razavi, 2020). Time and temperature of germination as well as legume type are the important factors that influence the production of antihypertensive peptides in pulses. Bamdad et al. (2009) demonstrated that by increasing germination time, more peptides with a lower MW and ACE inhibitory properties were produced in lentils. Mamilla and Mishra (2017) demonstrated that red lentil germination at 40°C could show a higher ACE inhibitory activity than sprouts obtained at 30°C. IC_50_ values for ACE activity obtained in this study are comparable to IC_50_ values of various intestinal digests of sprouted legumes listed in a recent review ranging from 0.2 to 0.8 mg/ml (Maleki and Razavi, 2020). As an example, in the literature, IC_50_ values for digested chickpea, cowpea, black pea, and white pea sprouts (3 days) of 0.6, 0.6, 0.7, and 0.3 mg/ml, respectively, are reported (Maleki and Razavi, 2020).

With respect to antioxidant activity, BL and ZF dry seeds showed the highest values of radical scavenging activity measured by ORAC (62.7 and 60.1 mmol TE/g, respectively) and ABTS methods (41.1 and 59.2 mmol TE/g, respectively) (Table 3). A higher antioxidant activity of these seed types as compared to GZL and DL could be associated with the presence of tannins (proanthocyanidins), main contributors to the antioxidant capacity although other polyphenols may also play a supporting role (Elessawy et al., 2021). Overall, antioxidant activity in the four legume samples increased gradually during digestion with the independence of germination treatment. Interestingly, ZF sprouts showed the highest antioxidant activity values for ORAC and ABTS methods (186.1 and 152.1 mmol TE/g, respectively).

In general, sprouting increases the antioxidant activity of legumes. Various studies report a significant increase in antioxidant activity in lentils (Aguilera et al., 2015), fava bean (Mekky et al., 2020), and pigeon pea (Sharma et al., 2019) when seeds were germinated from 1 to 10 days at 20–35°C. The higher antioxidant activity of sprouted seeds in comparison to dry seeds is attributed not only to the production of peptides but also to the accumulation of other antioxidant compounds like vitamins (E and C), polyphenols, and FAA (Lemmens et al., 2019). Plant-derived peptides and FAA such as Y, M, H, K, P, and W are accepted as antioxidants that act as metal-chelating and hydrogen-/electron-donating agents through interactions with free radicals, thereby terminating the radical chain reaction or preventing its formation (Matemu et al., 2021). Peptides may also exert antioxidant activity *via* certain amino acids although this property is determined by the amino acid sequence and composition (Nwachukwu and Aluko, 2019). When considering the differences observed in the protein, peptide, and FAA profiles, and seed microstructures after germination and during the gastrointestinal digestion of lentil and fava bean types, it is not surprising that the bioactivity changed from one pulse to another and from the gastric to the intestinal phase of digestion. Higher amounts of peptides and FAAs in intestinal digests could explain the powerful antioxidant effect of ZF sprouts at the end of the intestinal phase.

### Differences in the Peptidome of Intestinal Digests of Dry Seeds and Sprouts of Lentil and Fava Bean Types

Peptidome analyses were undertaken to explore the impact of germination and legume type on peptide profiles generated during digestion. The total number of peptides identified in the four legume samples before (GZL0, BL0, DL0, and ZF0) and after the germination for 96 h (GZL96, BL96, DL96, and ZF96) is shown in Supplementary Data 1. The method used in this study resulted in a window of identification of peptides between 814.46 and 4,172.18 Da to obtain an efficient fragmentation in the collision cell and a minimum peptide length of six amino acids that can be unambiguously matched to a single protein sequence. Some trends emerged when we compared the number of the identified peptides common to each sample and the median MW of the peptide population (Supplementary Figure 6). Focusing on a comparison between intestinal digests before germination, DL and ZF showed a lower number of peptides (39 and 68, respectively) and median MWs (1,323.6 and 1,421.1 Da, respectively). After germination, GZL and DL stood out with a comparable number of peptides (80 and 76, respectively) but a clear decrease in the median MW (1,285.1 and 1,190.5 Da, respectively) as compared to the intestinal digests of ungerminated seeds. These results led us to hypothesize that the proteins of GZL and DL sprouts are more rapidly and extensively digested than BL and ZF sprouts in agreement with conclusions drawn by other authors when comparing the intestinal peptidomes of milk proteins and enriched legume pasta (Berrazaga et al., 2020).

Most of the peptides in lentil intestinal digests belong to convicilin (≈30% of the total number of peptides) followed by allergen Len c 1.0101, allergen Len c 1.0102, and vicilin type C; whereas the main parent proteins in ZF were vicilin (40% of the total number of peptides) followed by legumin-type B and favin. The identified peptides were represented using the Peptigram web application (Supplementary Figure 7) that visualizes the digestion-resistant regions within a protein. The plots generated by Peptigram show the amino acids identified as part of a peptide sequence (vertical bars), the count of peptides overlapping this position (the height of bars), and a green color intensity that is proportional to peptide abundance. The blank areas in the plots correspond to protein regions completely degraded by the action of digestive enzymes. The first interesting observation was that allergen Len c 1.0101 and allergen Len c 1.0102 were more resistant to digestion than convicilin and vicilin in the three lentil types studied either before or after the germination (Supplementary Figures 7A–C). Similarly, vicilin in ZF showed more resistant regions to digestion than legumin-type BL and favin (Supplementary Figure 7D). By comparing lentil types, the main difference was observed for DL in which fewer resistant regions in the main storage proteins were found at the end of intestinal digestion. The absence of the seed coat in DL could have contributed to a higher digestibility of lentil storage proteins due to a rapid germination of this seed type that resulted in a more extensive seed endogenous proteolysis and the degradation of cell microstructure, favoring the diffusion of digestive enzymes (Zahir et al., 2021).

### Variations in the Bioactive Potential of Digestion-Resistant Peptides in Dry Seeds and Sprouts of Lentil and Fava Bean Seed Types

To identify bioactive peptide sequences in intestinal digests obtained from dry and sprouted lentils and fava bean, we used a combination of computational tools. At first, all identified peptide sequences in the intestinal digests of dry and sprouted legumes were processed with the Peptide Ranker that gave a range of score values from 0.0140 to 0.8654 (Supplementary Data 2). Peptides predicted with scores higher than 0.5 (threshold level set in the Peptide Ranker to label a peptide as bioactive) were selected and included in Supplementary Data 2. In total, 97 peptides released from convicilin, vicilin, allergen Len c 1.0101, allergen Len c 1.0102, legumin-type B, and favin were predicted to be bioactive and found to display two or more activities. The bioactivity that appeared with more frequency in all samples was ACE inhibitory activity (in consistency with the experimental results of Table 3) and dipeptidyl peptidase IV inhibitory activity. Moreover, the peptide sequence DLAIPVNRPGQL identified in DL sprouts has reported ACE inhibitory activity (García-Mora et al., 2017). In line with antioxidant activity shown by the intestinal digests of the studied legume types, a total of nine identified sequences (six in lentil and three in fava bean) were predicted to display antioxidant activity. In particular, the fava bean peptide LSPGDVLVIPAGYPVAIK shares the amino acid sequence VIPAGYP with an antioxidant peptide identified in fava bean hydrolysates (Samaei et al., 2020).

Differences in the number of bioactive peptides were observed among legume types and between dry and sprouted seeds (Supplementary Data 3). GZL and BL showed a higher number of bioactive peptides (21 and 20, respectively) that decreased after germination (7 and 12, respectively). A lower number of bioactive peptide sequences were found for DL (3) and ZF (7) although the germination of these legumes increased the number of bioactive sequences for DL (seven in total), whereas it remained unaltered for ZF. In the sprouts of GZL, BL, DL, and ZF, a total of 1, 4, 6, and 1 unique bioactive peptides were identified.

Absorption, distribution, metabolism, excretion, and transport (ADMET) properties are the crucial features of bioactive peptides. Therefore, BBB and HIA, as the two major factors of well-absorbed peptides, were predicted in this study using the AdmetSAR server. Herein, all predicted bioactive peptides had a high probability of permeability for BBB with values between 0.77 and 0.96 (Supplementary Data 2). In the prediction of HIA, 15 of 21 peptides in GZL, 15 of 24 in BL, 3 of 9 in DL, and 2 of 7 in ZF had the ability to be absorbed. These results indicate that a large amount of the identified peptides can be absorbed, showing a high probability to be bioavailable. Probability scores for AMES mutagenesis and carcinogenesis indicated that lentil and fava bean bioactive peptides are not mutagenic or carcinogenic. This observation is supported by a recent study, showing a high correlation between the ADMET predicted values and experimental results on the bioactivity and intestinal absorption of ACE inhibitory peptides (Zhao et al., 2019). Regarding hepatotoxicity, the peptides SDLFENL, LSPGDVFVVPAGHPVA, LSPGDVFVVPAGHPV, FLPQFTDADF in lentil and FFEITPK and ATPADVLANAFGL in fava bean showed low probability values (<0.6) that were smaller than those previously calculated for cocoa peptides (Coronado-Cáceres et al., 2020). The peptide SDLFENL identified in all lentil types and the peptide QQQPDSHQKIRR in ZF showed the lowest values of acute oral toxicity (2.1 and 2.3 kg/mol, respectively), whereas LSPGDVFVVPAGHPV found in GZL and BL lentils and LSPGDVLVIPAGYPVAIK in ZF exhibited the highest acute oral toxicity (3.4 and 3.2 kg/mol, respectively).

## Conclusions

This study provides significant insights into the role of the germination process in inducing distinct changes in the seed cellular structure, anti-nutrient profiles, as well as nutritional and healthy traits of lentil and fava bean proteins that are translated into an increment in their digestibility, the bio accessibility of peptides/amino acids, and the bioactivity of protein digestion end products. This study showed that seed permeability had an influence on the speed of the germination process and consequently on the degree of anti-nutrient and seed microstructure degradation and endogenous seed proteolysis, and ultimately in protein digestibility and the release of bioactive peptides. DL was characterized by a rapid imbibition rate, radicle emergence, and sprout development, which was associated with a greater cellular structure degradation, a lower anti-nutrient content, and a higher protein digestibility, and amino acid bioaccessibility as compared to the rest of the studied seed types. Although low tannin/zero vicine and convicine fava bean sprouts had a lower protein digestibility than lentil sprouts due to a slow germination, a higher anti-nutrient content, and a more intact cellular structure, this legume type showed a greater bioactive potential before and during the digestion. The intestinal phase of digestion was an important stage of peptide differentiation among seed types and between dry seeds and sprouts. Peptidome analysis revealed differences in the number of peptides, MW distribution, and bioactive profile of the nitrogen fraction and allowed the identification of protein regions resistant to gastrointestinal digestion. Several peptides resistant to gastrointestinal digestion were found to match with previously reported bioactive peptides, suggesting their potential health effects. Legume types with a higher protein digestibility showed a smaller number of total and bioactive peptides with a lower mean MW. In addition, lentil and fava bean vicilins were more resistant to digestion than legumins. This study provides new knowledge about the role of germination on protein digestibility and the bioactivity of pulses, suggesting the selection of specific types that could be employed to improve the nutritional value and health properties of foods.

## Data Availability Statement

The datasets presented in this study can be found in online repositories. The names of the repository/repositories and accession number(s) can be found below: http://www.ebi.ac.uk/pride, PXD027847.

## Author Contributions

JF, AV, EP, and CM-V: conceptualization. SB-E and EP: formal analysis and data curation. CM-V: project administration. SB-E and CM-V: writing—original draft. SB-E, AV, EP, JF, and CM-V: writing—reviewing and editing. AV: funding acquisition. All authors contributed to the article and approved the submitted version.

## Funding

This research was carried out with the financial support of the UoS grant Lentils for Sustainable and Healthy Proteins (Grant No. 20196324) through the SPG-NSERC Industrial Research Chair Program in Lentil Genetic Improvement. SB-E is a Postdoctoral Researcher funded by a grant Lentils for Sustainable and Healthy Proteins (Grant No. 20196324).

## Conflict of Interest

The authors declare that the research was conducted in the absence of any commercial or financial relationships that could be construed as a potential conflict of interest.

## Publisher's Note

All claims expressed in this article are solely those of the authors and do not necessarily represent those of their affiliated organizations, or those of the publisher, the editors and the reviewers. Any product that may be evaluated in this article, or claim that may be made by its manufacturer, is not guaranteed or endorsed by the publisher.

## Abbreviations

A: alanine
ACE: angiotensin converting enzyme
ABTS: 2,2′-azino-bis-3-ethylbenzothiazoline-6-sulfonic acid
ANOVA: One-way ANOVA
BL: beluga lentil
BBB: blood brain barrier
C: cysteine
CW: cell wall
D: aspartic acid
DH: degree of hydrolysis
DL: dehulled red lentil
E: glutamic acid
EAA: essential amino acids
F: phenylalanine
FAA: free amino acids
G: glycine
GZL: gray zero-tannin lentil
H: histidine
I: isoleucine
HIA: human intestinal absorption
K: lysine
L: leucine
M: methionine
N: asparagine
NEAA: non-essential amino acids
MW: molecular weight
ORAC: oxygen radical absorbance capacity
OPA: *o*-phthaldialdehyde
P: proline
PBS: phosphate buffer saline
PCA: principal component analysis
PSV: protein storage vacuoles
SDS-PAGE: sodium dodecyl sulfate-polyacrylamide gel electrophoresis
Q: glutamine
R: arginine
SIF: simulated intestinal fluid
SG: starch granules
SGF: simulated gastric fluid
SSF: simulated salivary fluid
T: threonine
TE: Trolox equivalents
TIA: trypsin inhibitory activity
TIU: trypsin inhibitory units
V: valine
UPLC-MS/MS: ultra-high performance liquid chromatography-tandem mass spectrometry
Y: tyrosine
ZF: low tannin/zero vicine–convicine fava bean.
