# Supplementary material for: Lentil and Fava Bean With Contrasting Germination Kinetics: A Focus on Digestion of Proteins and Bioactivity of Resistant Peptides
Source: Front Plant Sci. 2021 Oct 25;12:754287. doi: 10.3389/fpls.2021.754287 (PMC8575454; doi:10.3389/fpls.2021.754287)
Supplement: Supplementary file 1 [file Data_Sheet_1.zip › Supplementary Figures and Table.DOCX]

Supplementary Material

# Supplementary Figures and Tables

##
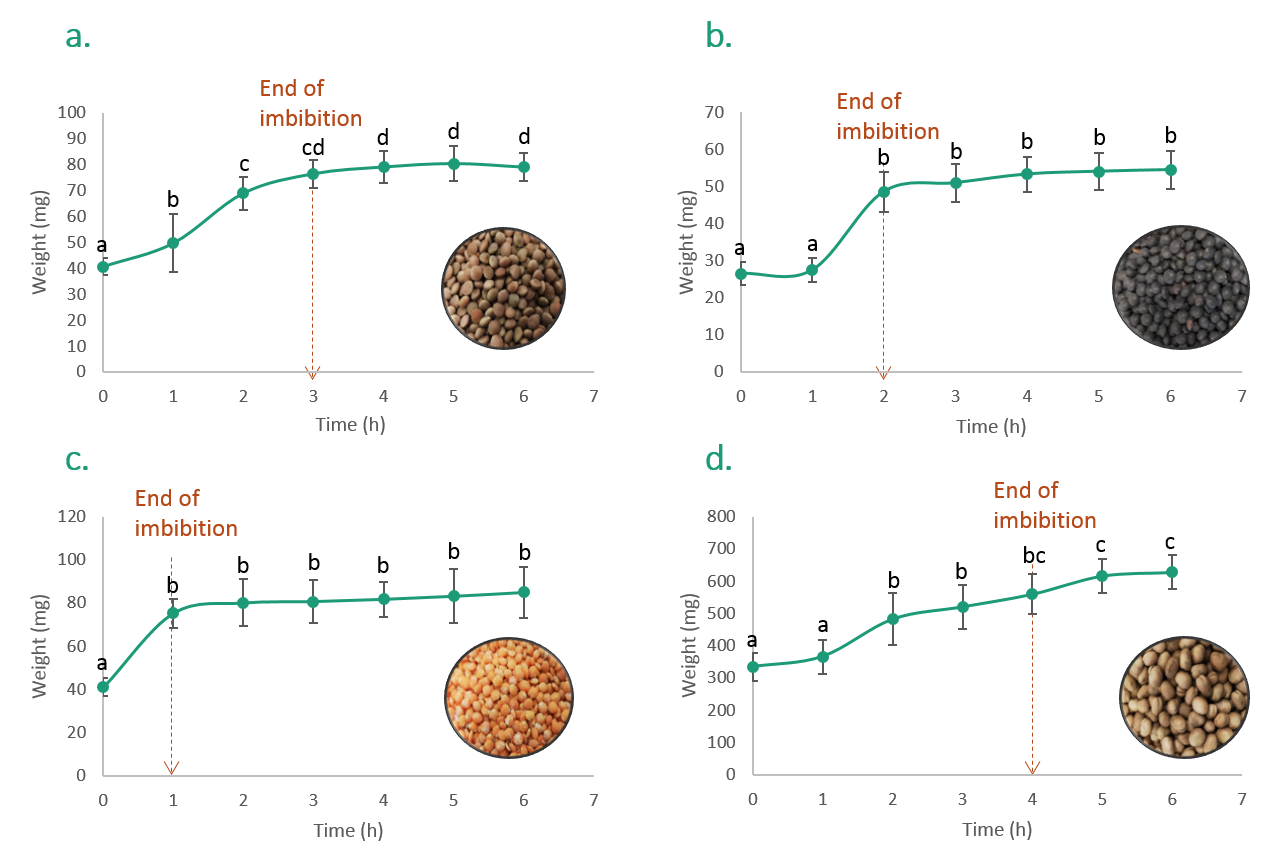
Supplementary Figures

**Supplementary Figure 1.** Seed weight gain of GZL (a), BL (b), DL (c) and ZF (d) at different soaking time points in water at 20 °C. Values are the mean and error bars represent the standard deviation of 5 biological replicates. Different letters indicate differences among mean values (p ≤ 0.05, Duncan *post hoc* test). Abbreviations: BL, beluga lentil; DL, red dehulled lentil; GZL, gray zero tannin lentil; ZF, low tannin/zero vicine-convicine fava bean.

**Supplementary Figure 2.** **.** Photographs of GZL (a), BL (b), DL (c) and ZF (d) at different times of germination. Radicle emergence was visible after 24 h for GZL, BL and ZF and after 15 h for DL. Abbreviations: BL, beluga lentil; DL, red dehulled lentil; GZL, gray zero tannin lentil; ZF, low tannin/zero vicine-convicine fava bean.

**Supplementary Figure 3.** Sprout length of GZL (a), BL (b), DL (c) and ZF (d) at different germination endpoints at 20 °C. Values are the mean and error bars represent the standard deviation of 5 biological replicates. Different letters indicate differences among mean values (*p* ≤ 0.05, Duncan post hoc test). Abbreviations: BL, beluga lentil; DL, red dehulled lentil; GZL, gray zero tannin lentil; ZF, low tannin/zero vicine-convicine fava bean.

**Supplementary Figure 4.** Changes in band intensity (expressed as relative % of dry seeds) of the major polypeptides tentatively identified by SDS-PAGE in GZL (a), BL (b), DL (c) and ZF (d) protein extracts. Optical density of individual bands was measured using Quantity-One software (Bio-rad, Hercules, CA, USA). Data represent the mean and standard deviation of three experimental replicates. Abbreviations: BL, beluga lentil; DL, red dehulled lentil; GZL, gray zero tannin lentil; ZF, low tannin/zero vicine-convicine fava bean

**
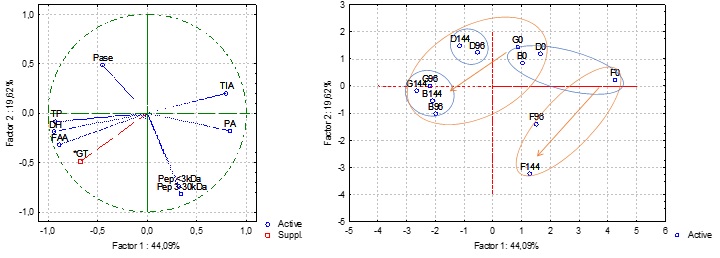
**

**Supplementary Figure 5.** Projection of variables (A) and cases (B) illustrating the principal component analysis for 4 legume types at different times of germination (0, 96 and 144 h) including with 10 markers of protein hydrolysis, protein and antinutrient content. Abbreviations: BL, beluga lentil; DL, red dehulled lentil; DH degree of hydrolysis; GZL, gray zero tannin lentil; ZF, low tannin/zero vicine-convicine fava bean; FAA, free amino acids, GT, germination time; PA, Phytic acid Pase, protease activity; Pep<3kDa, peptides below 3 kDa; Pep3-30kDa, peptides between 3 to 30 kDa; TIA, trypsin inhibitory activity; TP, total protein.

**Supplementary Figure 6.** Boxplot of the molecular weights of peptides identified after 120 min of intestinal digestion of raw seeds (GZL0, BL0, DL0 and ZF0) compared to germinated seeds for 96 h (GZL96, BL96, DL96 and ZF96). Table summarizes the number of peptide sequences and median molecular weight in intestinal digests for each type of legume. Abbreviations: BL, beluga lentil; DL, red dehulled lentil; GZL, gray zero tannin lentil; ZF, low tannin/zero vicine-convicine fava bean.

**Supplementary Figure 7.** Gastrointestinal digestion resistant peptides from major proteins present in raw (sample codes including 0) and sprouted (sample codes including 96) legumes [GZL (panel a), BL (panel b), DL (panel c) and ZF (panel d)] were plotted using the Peptigram Bioware tool. Each vertical bar corresponds to an amino acid identified as part of a peptide sequence. The height of the bar is proportional to the number of the peptides overlapping this position and the colour intensity is proportional to the sum of intensities of the peptides overlapping a given position. Abbreviations: BL, beluga lentil; DL, red dehulled lentil; GZL, gray zero tannins lentil; ZF, low tannin/zero vicine-convicine fava bean.

## Supplementary Tables

**Supplementary Table 1.** Germination rate, dry matter and moisture content of lentil and fava bean seeds germinated at 20 °C for 96 and 144 h after the end of imbibition phase.

|  | Time (h) | GZL | BL | DL | ZF |
| --- | --- | --- | --- | --- | --- |
| Germination Rate (%) | 96 | 97.33 ± 2.31 ^a^ | 95.33 ± 4.16 ^a^ | 94.00 ± 2.00 ^a^ | 99.33 ± 1.15 ^a^ |
|  | 144 | 100.0 ± 0.0 ^a^ | 100.0 ± 0.0 ^a^ | 99.33 ± 1.15 ^b^ | 100.0 ± 0.0 ^a^ |
| Dry Weight (%) | 96 | 40.81 ± 4.36 ^a^ | 41.60 ± 1.21 ^a^ | 41.51 ± 1.38 ^a^ | 40.66 ± 1.01 ^a^ |
|  | 144 | 38.64 ± 1.58 ^a^ | 45.56 ± 1.89 ^a^ | 37.72 ± 3.47 ^a^ | 39.18 ± 1.55 ^a^ |
| Moisture (%) | 96 | 59.19 ± 4.36 ^a^ | 58.40 ± 1.21 ^a^ | 58.49 ± 1.38 ^a^ | 59.34 ± 1.01 ^a^ |
|  | 144 | 61.35 ± 1.58 ^a^ | 54.43 ± 1.89 ^a^ | 63.27 ± 5.47 ^a^ | 60.81 ± 1.56 ^a^ |

Values are the mean ± standard deviation of three experimental replicates. Different letters indicate differences among mean values of each variable (*p* ≤ 0.05, Duncan *post hoc* test). Abbreviations: BL, beluga lentil; DL, red dehulled lentil; GZL, gray zero tannins lentil; ZF, low tannin/zero vicine-convicine fava bean.
